# The Association of Tobacco Smoking, Second-hand Smoke, and Novel Tobacco Products With COVID-19 Severity and Mortality in Italy: Results From the COSMO-IT Study

**DOI:** 10.2188/jea.JE20220321

**Published:** 2023-07-05

**Authors:** Silvano Gallus, Cristina Bosetti, Giuseppe Gorini, Chiara Stival, Roberto Boffi, Alessandra Lugo, Giulia Carreras, Chiara Veronese, Claudia Santucci, Roberta Pacifici, Biagio Tinghino, Vincenzo Zagà, Patrizia Russo, Maria Sofia Cattaruzza, the COSMO-IT Investigators

**Affiliations:** 1 Department of Environmental Health Sciences, Istituto di Ricerche Farmacologiche Mario Negri IRCCS, Milan, Italy; 2 Department of Oncology, Istituto di Ricerche Farmacologiche Mario Negri IRCCS, Milan, Italy; 3 Oncologic Network, Prevention and Research Institute (ISPRO), Florence, Italy; 4 Pulmonology Unit, Fondazione IRCCS Istituto Nazionale dei Tumori, Milan, Italy; 5 Department of Clinical Sciences and Community Health, University of Milan, Milan, Italy; 6 National Center of Addiction and Doping, Istituto Superiore di Sanità, Rome, Italy; 7 Tobacco Unit, Department of Mental Health and Addiction, ASST Brianza, Vimercate, Italy; 8 Società Italiana di Tabaccologia (SITAB), Rome, Italy; 9 Department of Human Sciences and Quality of Life Promotion San Raffaele University, Rome, Italy; 10 Clinical and Molecular Epidemiology, IRCCS San Raffaele Roma, Rome, Italy; 11 Department of Public Health and Infectious Diseases, Sapienza University, Rome, Italy

**Keywords:** COVID-19, electronic cigarettes, heat-not-burn tobacco, heated tobacco products, prognosis, severity, mortality, tobacco smoking, second-hand smoke

## Abstract

**Background:**

Despite the robust evidence of an excess risk of coronavirus disease 2019 (COVID-19) severity and mortality in ever smokers, the debate on the role of current and ex-smokers on COVID-19 progression remains open. Limited or no data are available on the link between electronic cigarette (e-cigarette), heated tobacco product (HTP) and second-hand smoke (SHS) exposure and COVID-19 progression. To fill this knowledge gap, we undertook the COvid19 and SMOking in ITaly (COSMO-IT) study.

**Methods:**

A multi-centre longitudinal study was conducted in 2020–2021 in 24 Italian hospitals on a total of 1,820 laboratory-confirmed COVID-19 patients. We estimated multivariable odds ratios (OR) and 95% confidence intervals (CI) to quantify the association between smoking-related behaviours (ie, smoking status, e-cigarette and HTP use, and SHS exposure) and COVID-19 severity (composite outcome: intubation, intensive care unit admission and death) and mortality.

**Results:**

Compared to never smokers, current smokers had an increased risk of COVID-19 mortality (OR 2.17; 95% CI, 1.06–4.41). E-cigarette use was non-significantly associated to an increased risk of COVID-19 severity (OR 1.60; 95% CI, 0.96–2.67). An increased risk of mortality was observed for exposure to SHS among non-smokers (OR 1.67; 95% CI, 1.04–2.68), the risk being particularly evident for exposures of ≥6 hours/day (OR 1.99; 95% CI, 1.15–3.44).

**Conclusion:**

This multicentric study from Italy shows a dismal COVID-19 progression in current smokers and, for the first time, in SHS exposed non-smokers. These data represent an additional reason to strengthen and enforce effective tobacco control measures and to support smokers in quitting.

## INTRODUCTION

Data from the literature have shown that male sex, advanced age, and presence of comorbidities are the major determinants of a poor prognosis for coronavirus disease 2019 (COVID-19), an infectious disease caused by the novel SARS-CoV-2 coronavirus that spread around the world in early 2020.^1^^–^^3^

Since the beginning of the pandemic, tobacco smoking has also been hypothesized to be associated with COVID-19 progression (ie, severity and/or mortality), given that tobacco smoking was found to be linked with other (similar) respiratory infectious diseases and lungs are the organs mostly affected by COVID-19.^3^^–^^5^ Longitudinal prospective studies on COVID-19 patients found that smokers had a substantial increased risk of major unfavorable health outcomes, including hospitalizations in an intensive care unit (ICU), need for mechanical ventilation, or death, with an about 30–45% increased risk of disease progression.^3^^,^^6^^–^^11^ However, despite the relatively high number of cohort studies on the role of tobacco smoking on COVID-19 outcomes, the majority of such studies did not provide adjusted risk estimates.^7^

Second-hand smoke (SHS) involves the exposure to many of the toxic substances inhaled during active smoking, thus it is reasonable to suspect that SHS may also play a role on COVID-19 outcomes. To the best of our knowledge, however, the evidence on the association between SHS exposure and COVID-19 severity and mortality is limited to a small study on pediatric patients positive to SARS-CoV-2, showing more frequent symptoms among minors exposed to SHS.^12^^,^^13^ With reference to tobacco products other than conventional tobacco cigarettes—such as electronic cigarettes (e-cigarette) and heated tobacco products (HTPs)—there is a large gap of knowledge.^14^ A few cross-sectional and cohort studies showed that e-cigarette users have more frequent and persistent COVID-19 symptoms,^15^^,^^16^ but only a recent cohort study analyzed the role of e-cigarettes on COVID-19 severity, and the study did not find any relationship between e-cigarette use and COVID-19 hospitalization and mortality.^17^ To our knowledge, no study has been conducted so far on the role of HTPs on COVID-19 severity and mortality.

In order to further investigate and quantify the effect of tobacco smoking and exposure to other novel products and SHS on COVID-19 severity and mortality, we conducted a large prospective study on patients hospitalized for COVID-19 in 24 Italian centers (the COvid19 and SMOking in ITaly, COSMO-IT, study).^18^

## METHODS

### Study patients

COSMO-IT is an observational, longitudinal, multicentric study of 1,820 laboratory-confirmed COVID-19 patients hospitalized between March 2020 and April 2021 in 24 Italian centers from northern, central, and southern Italy.^18^ Patients were consecutively identified in various hospital wards mainly coordinated by pneumologists collaborating within the Società Italiana di Tabaccologia, Rome, Italy.

The study protocol was approved by the Ethics Committee of the Coordinating centre (Comitato Etico Regionale Toscana Area Vasta Centro, N. 17495) and subsequently approved by the Ethics Committees of all the participating centers. All patients included in the study provided an informed consent to participate; for deceased patients, the informed consent was obtained from a familiar or a close relative.

### Data collection

For each patient enrolled in the study, we collected data on sociodemographic characteristics, major pre-existing chronic conditions, prior use of selected drugs, anthropometric factors, and selected lifestyle habits. In particular, we collected information on smoking status (ie, never, ex- and current smoker), current daily exposure to SHS (among non-smokers), and ever use of e-cigarettes or HTPs (ie, current regular, current occasional, and past use). Information on COVID-19 symptoms, date at onset, severity of the disease, type of hospitalization, therapy, prescribed drugs, and possible complications during hospitalization were also collected. At the end of hospitalization, the outcome of the disease was recorded: discharge or laboratory-confirmed recovery, any transfer, or death. Baseline and follow-up information were collected through a questionnaire for patients enrolled prospectively; for patients hospitalized before the Ethics Committees’ approval and enrolled retrospectively, data were extracted from patients’ medical records (whenever available) or obtained by questionnaire directly contacting patients (or their relatives, in case of deceased patients).

Following previous studies,^7^ for COVID-19 severity we considered a composite outcome, defined as intubation, admission to ICU or death (when available); death was also analyzed among 1,129 patients from 15 centers which were able to collect information on patients’ vital status.

### Statistical analysis

Data were presented as absolute and relative frequencies, for categorical variables, or means and standard deviations (SD), for continuous ones. The association between various smoking exposures and the risk of COVID-19 severity and prognosis was quantified using univariable logistic regression models to estimate the odds ratios (ORs) and corresponding 95% confidence intervals (CIs). Multivariable ORs were also estimated, after allowance for sex, age, education, and cigarette smoking. Additional OR adjusted for body mass index were also provided.

## RESULTS

Among 1,820 patients included in the analysis, 37.9% were women, the mean age was 64.4 (SD, 15.1) years, and 19.1% had a low level of education; corresponding figures were 39.3%, 66.2 (SD, 15.6) years, and 22.1% among 1,129 patients from 15 centers with information on patients’ vital status (eTable 1).

Overall, the composite outcome (ie, intubation, admission to ICU or death) occurred in 19.2% of patients, was more frequent among men than women (21.3% vs 15.8%; *P* = 0.004) and among patients aged ≥80 years than those aged <50 years (32.7% vs 7.2%; *P* for trend ≤0.001; eTable 2). Among 1,129 patients from 15 centers which provided information on patients’ vital status, 14.8% deceased, with a similar frequency in men and women (14.9% vs 14.6%; *P* = 0.908), but a much higher frequency in older than younger patients (37.4% vs 2.3%; *P* for trend <0.001).

Table 1 shows univariable and multivariable ORs of composite outcome and death, according to tobacco smoking and use of e-cigarettes and HTPs. Univariable analyses did not show any association, except for a statistically significant increase of COVID-19 severity for ex-smokers compared to never smokers. After allowance for age, sex, education, and cigarette smoking, these associations were no longer statistically significant. A borderline significant increased risk in COVID-19 severity was observed for ever e-cigarette users compared to never e-cigarette users (adjusted OR 1.60; 95% CI, 0.96–2.67). With reference to COVID-19 mortality, a significant increased risk was found for current smokers compared to never smokers (OR 2.17; 95% CI, 1.06–4.41). Such estimate was 1.89 (95% CI, 0.91–3.93) after adjusting also for e-cigarette use and HTP use, and 2.25 (95% CI, 1.07–4.72) after exclusion of e-cigarette and HTP users. No significant associations with mortality were found for e-cigarette (OR 1.24) and HTP (OR 1.16) ever users compared to never users. Further adjustment for body mass index did not meaningfully modify the risk estimates (eTable 3).

**Table 1.** Univariable and multivariable odds ratios of composite outcome and death according to various smoking exposures among patients hospitalized for COVID-19, Italy, 2020–2021

	*N* ^c^	Composite outcome^a^			*N* ^c^	Death^b^		
	
		%	Crude OR (95% CI)^d^	Adjusted OR (95% CI)^e^		%	Crude OR (95% CI)^d^	Adjusted OR (95% CI)^e^
Cigarette smoking								
Never smokers	907	17.1	1.00^f^	1.00^f^	562	13.3	1.00^f^	1.00^f^
Ex-smokers	787	22.4	1.40 (1.10–1.78)	1.21 (0.94–1.58)	476	16.4	1.27 (0.90–1.79)	1.22 (0.82–1.83)
Current smokers	126	15.1	0.86 (0.51–1.45)	0.96 (0.56–1.64)	91	15.4	1.18 (0.64–2.19)	2.17 (1.06–4.41)

E-cigarette								
Never users	1,675	19.3	1.00^f^	1.00^f^	1,055	14.9	1.00^f^	1.00^f^
Ever users	98	24.5	1.36 (0.84–2.19)	1.60 (0.96–2.67)	66	15.2	1.02 (0.51–2.05)	1.24 (0.56–2.72)

Heated tobacco products								
Never users	1,715	19.7	1.00^f^	1.00^f^	1,093	14.8	1.00^f^	1.00^f^
Ever users	29	20.7	1.07 (0.43–2.64)	1.21 (0.47–3.12)	26	15.4	1.05 (0.36–3.07)	1.16 (0.35–3.89)

CI, confidence interval; COVID-19, coronavirus disease 2019; OR, odds ratio.

^a^Defined as intubation, admission to intensive care unit, or death; based on 1,820 patients.

^b^Based on 1,129 patients.

^c^The sums do not add to the total because of a few missing values.

^d^Estimates from univariable logistic regression models.

^e^Estimates from multivariable logistic regression models, adjusted for sex, age, and level of education. Estimates for e-cigarette and heated tobacco products are also adjusted for smoking.

^f^Reference category.

An increased risk of mortality was observed for exposure to SHS (OR 1.67; 95% CI, 1.04–2.68). The risk for SHS was particularly evident for exposures of 6 or more hours a day compared to non-exposed patients (OR 1.99; 95% CI, 1.15–3.44; Table 2). Estimates were similar after further allowance for body mass index (eTable 3).

**Table 2.** Univariable and multivariable odds ratios of composite outcome and death according to second-hand smoke among no smoking patients hospitalized for COVID-19, Italy, 2020–2021

	*N* ^c^	Composite outcome^a^			*N* ^c^	Death^b^		
	
		%	Crude OR (95% CI)^d^	Adjusted OR (95% CI)^e^		%	Crude OR (95% CI)^d^	Adjusted OR (95% CI)^e^
Second-hand smoke daily exposure								
No	992	19.2	1.00^f^	1.00^f^	680	11.8	1.00^f^	1.00^f^
Yes	335	18.5	1.17 (0.81–1.68)	0.96 (0.69–1.33)	231	17.3	1.57 (1.04–2.37)	1.67 (1.04–2.68)
Yes, <6 hours/day	157	15.3	0.76 (0.48–1.21)	0.85 (0.53–1.37)	93	10.8	0.90 (0.45–1.81)	1.16 (0.53–2.51)
Yes, ≥6 hours/day	178	21.3	1.15 (0.77–1.70)	1.04 (0.69–1.57)	138	21.7	2.08 (1.31–3.32)	1.99 (1.15–3.44)
*P* for trend			0.798	0.985			0.005	0.016

CI, confidence interval; COVID-19, coronavirus disease 2019; OR, odds ratio.

^a^Defined as intubation, admission to intensive care unit, or death; based on 1,820 patients.

^b^Based on 1,129 patients.

^c^The sums do not add to the total because of a few missing values.

^d^Estimates from univariable logistic regression models.

^e^Estimates from multivariable logistic regression models, adjusted for sex, age, and level of education.

^f^Reference category.

## DISCUSSION

The present study on patients hospitalized for COVID-19 found an over two-fold excess risk of death in current smokers and a 67% increased risk of COVID-19 mortality for patients daily exposed to SHS.

Our study confirms the dismal prognosis of COVID-19 in current smokers. These findings update and confirm results from previous meta-analyses showing an excess risk of death for smokers compared to never smokers.^7^^–^^10^^,^^19^^–^^22^ In our study, however, the association with COVID-19 mortality was more modest and not significant in ex-smokers. This appears somewhat in contrast with the findings from other studies reporting a higher excess risk in ex-smokers.^7^^,^^10^ The meta-analysis by Simons and colleagues reported a significant relative risk (RR) for ex- versus never smokers (RR 1.39; 95% credible interval, 1.09–1.87), while it found a non-significant association for current versus never smokers (RR 1.22; 95% credible interval, 0.78–1.94).^7^ Such discrepancy can be explained by the fact that current smokers among COVID-19 patients are much younger than never and particularly former smokers^23^ and age is by far the strongest risk factor for COVID-19 severity and, particularly, mortality.^24^ For example, in our population mean age was 58.3 years among current smokers, 66.6 years among ex-smokers, and 63.3 years among never smokers (eTable 4). Considering that around 80% of currently available studies provided only crude risk estimates of the association between tobacco smoking and COVID-19 mortality, it is likely that the RR for current smokers is under-estimated and that for former smokers over-estimated in those studies not providing adjusted estimates by age.

To our knowledge, this is the first study providing data on the role of SHS exposure on COVID-19 progression. We found that, among COVID-19 non-smoking patients, daily exposure to SHS was directly related to death. However, tobacco smoking and daily SHS were not found to be associated with COVID-19 severity.

With reference to novel nicotine containing products, we observed a non-significant 60% increased risk of COVID-19 severity for use of e-cigarettes. Our data suggest a possible unfavorable effect of these products on COVID-19 progression, although some residual confounding by tobacco smoking may explain the associations observed. In the literature data on these products and COVID-19 severity/mortality are extremely limited: only a recent cohort study analyzed the role of e-cigarettes on COVID-19 severity, reporting no association with mortality (RR 1.12; 95% CI, 0.81–1.55),^17^ while no cohort analyzed the role of HTPs.

The present study has various strengths, but also limitations. To our knowledge, this is the first Italian longitudinal study based on a large multicenter cohort of patients hospitalized for COVID-19. Moreover, it was specifically designed to quantify the association between various smoking exposures and COVID-19 prognosis. In most previous cohort studies, smoking data are secondary; in other words, they have not been designed with the primary aim to quantify the association between smoking and COVID-19 outcomes. Further, we presented estimates adjusted for age (besides sex and education, which are other two relevant confounding factors), while most previous studies provided only crude RR, that—as discussed above—may provide biased estimates. Among the limitations of our study, there is the fact that not all the information on cigarettes and other smoking products was available from the medical records, and in various centers it was not possible to retrieve information for deceased patients. Moreover, our study sample did not allow to conduct specific stratified analyses to analyze the association of tobacco smoking with COVID-19 progression by use of novel products.

The excess risk of mortality in current smokers compared to never smokers suggests that a non-negligible proportion of the COVID-19 deaths may be attributable to tobacco smoking. Therefore, governments and policy makers have an additional reason to strengthen and enforce effective tobacco control measures and to support smokers in quitting.

## SUPPLEMENTARY MATERIAL

eTable 1. Characteristics of 1,820 patients hospitalized for COVID-19, Italy, 2020–2021

eTable 2. Distribution of patients hospitalized for COVID-19 according to a composite outcome and death, overall, by sex and age, Italy, 2020–2021

eTable 3. Multivariable odds ratios of composite outcome and death according to various smoking exposures further adjusted for body mass index among patients hospitalized for COVID-19, Italy, 2020–2021

eTable 4. Distribution of 1,820 patients hospitalized for COVID-19 by sex and age, according to various smoking exposures. Italy, 2020–2021
