# Supplementary material for: The Association of Tobacco Smoking, Second-hand Smoke, and Novel Tobacco Products With COVID-19 Severity and Mortality in Italy: Results From the COSMO-IT Study
Source: J Epidemiol. 2023 Jul 5;33(7):367–71. doi: 10.2188/jea.JE20220321 (PMC10257992; doi:10.2188/jea.JE20220321)
Supplement: Supplementary file 1 [file je-33-367-s001.pdf]

eTable 1. Characteristics of 1,820 patients hospitalized for COVID-19, Italy, 2020–2021

	**All patients (N=1,820)**	**Patients with information on vital status (N=1,129)**
	**N (%)**	**N (%)**
Sex		
Female	689 (37.9)	444 (39.3)
Male	1,131 (62.1)	685 (60.7)
Age, years		
<50	292 (16.0)	173 (15.3)
50–59	415 (22.8)	226 (20.0)
60–69	425 (23.4)	245 (21.7)
70–79	364 (20.0)	220 (19.5)
≥80	324 (17.8)	265 (23.5)
Mean (SD)	64.4 (15.1)	66.2 (15.6)
Education		
None/Elementary school	348 (19.1)	249 (22.1)
Middle school	522 (28.7)	324 (28.7)
High school	620 (34.1)	375 (33.2)
University	270 (14.8)	138 (12.2)
Other	60 (3.3)	43 (3.8)

COVID-19, novel coronavirus disease 2019; SD, standard deviation.

eTable 2. Distribution of patients hospitalized for COVID-19 according to a composite outcome and death, overall, by sex and age, Italy, 2020–2021

**Characteristics**	**Overall**		**Sex (%)**			**Age, years, (%)**					
	**N**	**%**	**Men**	**Women**	***P*-value**	**<50**	**50–59**	**60–69**	**70–79**	**≥80**	***P* for trend**
Composite outcome[a]											
No	1,470	80.8	78.7	84.2	0.004	92.8	84.3	79.8	80.2	67.3	<0.001
Yes	350	19.2	21.3	15.8		7.2	15.7	20.2	19.8	32.7	
Vital status[b]											
Alive	962	85.2	85.1	85.4	0.908	97.7	95.1	93.1	83.6	62.6	<0.001
Dead	167	14.8	14.9	14.6		2.3	4.9	6.9	16.4	37.4	

COVID-19, novel coronavirus disease 2019.
[a]Defined as intubation, admission to intensive care unit or death; based on 1,820 patients.
[b]Based on 1,129 patients from 15 centres.

eTable 3. Multivariable odds ratios of composite outcome and death according to various smoking exposures further adjusted for body mass index among patients hospitalized for COVID-19, Italy, 2020–2021

	**Composite outcome[a]**	**Death[b]**
	**Adjusted OR (95% CI)[c]**	**Adjusted OR (95% CI)[c]**
Tobacco smoking		
Never smokers	1.00[d]	1.00[d]
Ex-smokers	1.19 (0.91–1.54)	1.18 (0.79–1.77)
Current smokers	0.92 (0.54–1.58)	2.10 (1.03–4.28)
E-cigarette		
Never users	1.00[d]	1.00[d]
Ever users	1.58 (0.95–2.65)	1.22 (0.55–2.70)
Heated tobacco products		
Never users	1.00[d]	1.00[d]
Ever users	1.22 (0.47–3.16)	1.17 (0.35–3.91)
Second-hand smoke daily exposure[e]		
No	1.00[d]	1.00[d]
Yes	0.95 (0.68–1.32)	1.63 (1.01–2.63)
Yes, <6 hours/day	0.87 (0.54–1.41)	1.16 (0.53–2.55)
Yes, 6+ hours/day	1.01 (0.67–1.53)	1.93 (1.11–3.35)
P for trend	0.884	0.023

CI, confidence interval; COVID-19, novel coronavirus disease 2019; OR, odds ratio.
[a]Defined as intubation, admission to intensive care unit, or death; based on 1820 patients.
[b]Based on 1129 patients.
[c]Estimates from multivariable logistic regression models, adjusted for sex, age, level of education, and body mass index. Estimates for e-cigarette and heated tobacco products are also adjusted for smoking.
[d]Reference category.
[e]Among never and ex-smokers smokers; estimates not adjusted for cigarette smoking.

eTable 4. Distribution of 1,820 patients hospitalized for COVID-19 by sex and age, according to various smoking exposures. Italy, 2020–2021

	N[a]	Male sex (%)	Age, years Mean (SD)
Tobacco smoking			
Never	907	52.3	63.3 (16.7)
Ex	787	72.9	66.6 (12.5)
Current	126	65.9	58.3 (15.0)
Second-hand smoke[b]			
No	992	62.9	65.2 (15.1)
Yes	335	63.3	64.8 (15.2)
E-cigarette			
Never	1,675	62.5	64.7 (14.9)
Ever	98	60.2	61.2 (15.2)
Heated tobacco products			
Never	1,715	62.6	64.5 (15.0)
Ever	29	55.2	62.4 (17.6)

COVID-19, novel coronavirus disease 2019; SD, standard deviation.
[a]The sums do not add to the total because of a few missing values.
[b]Among never of ex-smokers only.
